# Surface Hydrophilic Modification of Polypropylene by Nanosecond Pulsed Ar/O_2_ Dielectric Barrier Discharge

**DOI:** 10.3390/ma18010095

**Published:** 2024-12-29

**Authors:** Yang Zhou, Zhi Fang, Yi Zhang, Tingting Li, Feng Liu

**Affiliations:** 1 College of Electrical Engineering and Control Science, Nanjing Tech University, Nanjing 211816, China; zy15251707128@163.com (Y.Z.); myfz@263.net (Z.F.); tingtingli0403@163.com (T.L.); 2 School of Energy Science and Engineering, Nanjing Tech University, Nanjing 211816, China

**Keywords:** polypropylene, hydrophilicity, surface modification, dielectric barrier discharge, oxygen

## Abstract

Polypropylene (PP) membranes have found diverse applications, such as in wastewater treatment, lithium-ion batteries, and pharmaceuticals, due to their low cost, excellent mechanical properties, thermal stability, and chemical resistance. However, the intrinsic hydrophobicity of PP materials leads to membrane fouling and filtration flux reduction, which greatly hinders the applications of PP membranes. Dielectric barrier discharge (DBD) is an effective technique for surface modification of materials because it generates a large area of low-temperature plasma at atmospheric pressure. In this study, O_2_ was added to nanosecond pulsed Ar DBD to increase its reactivity. Electrical and optical diagnostic techniques were used to study the discharge characteristics of the DBD at varying O_2_ contents. The uniformity of the discharge was quantitatively analyzed using the observed discharge images. Water contact angle measurements were used to assess the surface hydrophilicity of polypropylene. The surface morphology and chemical composition of the PP materials before and after treatment were analyzed using field emission scanning electron microscopy (FE-SEM), atomic force microscopy (AFM), Fourier-transform infrared spectroscopy (FTIR), and X-ray photoelectron spectroscopy (XPS). The results show that the moderate addition of O_2_ enhances surface hydrophilicity and the uniformity of the modification. By increasing the O_2_ addition from 0% to 0.1%, the average power increased from 4.19 W to 5.79 W, and the energy efficiency increased from 17.78% to 21.51%. The water contact angle of the DBD-treated PP showed a tendency to decrease and then increase with increasing O_2_ content, with the optimum O_2_ addition determined to be 0.1%. Under this condition, the water contact angle of the PP surface decreased by 31.88°, which is 52.31% lower than the untreated surface. O_2_ increases the number of oxygen-containing polar groups (-OH, C=O, and O-C=O) on the surface of the material, and deepens and densifies the grooves on the surface of the PP material, resulting in an increase in the hydrophilicity of the PP surface.

## 1. Introduction

Polypropylene (PP) is a versatile polymer widely used in various industries, such as wastewater treatment, automotive, food, electronics, and electrical appliances, due to its attractive mechanical characteristics, good thermal stability, and high resistance to chemicals [1,2,3,4]. However, constrained by its surface hydrophilicity and deficient adhesive properties, PP is susceptible to issues like membrane fouling, interfacial cracking, material aging, and performance degradation [5]. To overcome these challenges, hydrophilic modification is essential to broaden its application spectrum. Currently, the main techniques for surface modification are flame and heat treatment [6], graft copolymerization [7], grinding and physical treatment [8], and the plasma method [9]. The plasma method can effectively improve the hydrophilicity of polymers by changing the physical and chemical properties of the material surface in a depth range from a few nanometers to a few hundred nanometers, according to the modification requirements, such as with etching and the introduction of oxygen-containing polar groups [10,11,12]. Compared to other modification methods, the plasma method offers advantages such as low cost, ease of operation, high reliability, and environmental friendliness [13,14].

Common types of low-temperature plasma at atmospheric pressure include corona discharge [15], glow discharge [16], jet discharge [17], arc discharge [18], and dielectric barrier discharge (DBD) [19]. Among them, DBD is widely used in the field of polymer surface modification due to its ability to generate large-area plasma with moderate energy density at atmospheric pressure [20,21,22]. However, at atmospheric pressure, the electron mean free path is short, resulting in DBD being dominated by a filamentary discharge mode. This can lead to uneven surface modifications and even burning of the material surface [23,24]. To enhance the uniformity of DBD at atmospheric pressure, noble gases such as argon (Ar) and helium (He) are commonly used. These gases reduce the breakdown field strength and minimize the spatial electric field distortion [25]. However, the use of pure noble gases in DBD typically yields only ions and excited state particles, limiting the chemical activity and variety of active species. This inadequacy fails to meet the requirements for highly active plasma in the numerous practical applications of DBD.

To overcome this limitation and improve the effectiveness of hydrophilic modification treatments on DBD materials, researchers have introduced small amounts of reactive gases (O_2_, H_2_O, NH_3_, etc.) into a single inert gas. Lin et al. [26] demonstrated that the addition of H_2_O to the working gas effectively generates reactive particles such as OH particles, improving DBD discharge uniformity and plasma activity. Sauerbier et al. [27] used different working gases (Ar/O_2_, Ar/N_2_, and synthetic air) in a DBD apparatus to treat polypropylene-based wood–plastic composites. They found that materials treated with Ar/O_2_ and Ar/N_2_ were rougher, had a significantly reduced water contact angle, and underwent better surface modification compared to synthetic air.

Among the added gases, O_2_ stands out for its ability to produce oxidizing radicals such as the O atom, which accelerates the surface oxidative decomposition of polymer materials, induces an etching effect, and forms oxygen-containing polar groups. For example, Zheng et al. [28] modified polyethylene terephthalate (PET) using Ar/O_2_ plasma, introducing polar groups and increasing the surface roughness. Pappas et al. [29] observed the increased hydrophilicity of polymer surfaces after treatment with He/O_2_ DBD. Fang et al. [30] found that the addition of small amounts of O_2_ to Ar DBD for the surface treatment of PET significantly enhanced hydrophilicity, with the optimum effect observed at 0.3% O_2_.

In this study, O_2_ was added to a nanosecond pulsed Ar DBD and electrical and optical diagnostic techniques were used to diagnose the discharge characteristics at different O_2_ contents. Water contact angle measurements were used to assess the surface hydrophilicity of PP, and the physical morphology and chemical composition of PP were examined using scanning electron microscopy (SEM), atomic force microscopy (AFM), attenuated total reflectance Fourier-transform infrared spectroscopy (FTIR), and X-ray photoelectron spectroscopy (XPS). The results of the water contact angle measurements show that the moderate addition of O_2_ to nanosecond pulsed Ar DBD improves the surface hydrophilicity of PP. The research findings provide important reference data for optimizing the experimental conditions and assessing the effectiveness of surface modification of DBD polymer materials with nanosecond Pulsed Ar/O_2_.

## 2. Experimental

### 2.1. DBD Plasma Generation

**Figure 1:**
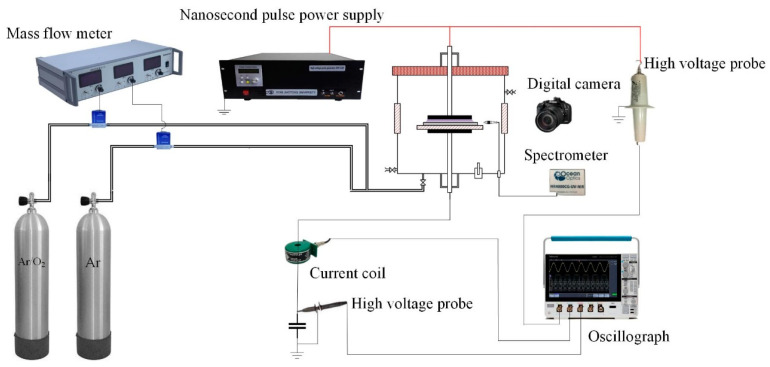
Experimental setup and measurement system of DBD surface modification.

The experimental setup and measurement system used in this study are shown in Figure 1. The experimental system consisted of a nanosecond pulse power supply (Xi’an HV-2015 Xi’an High Voltage, Xi’an, China), a DBD reactor (Self-design), and an electrical and optical diagnosis system. The voltage amplitude in the experiment was set to 4.2 kV, the repetition frequency was 4 kHz, the pulse width was 1000 ns, and the time of the rising and falling edges of the pulse was 50 ns. The DBD reactor was surrounded by quartz observation windows, which were convenient for observing and photographing the discharge images. The interior was made of stainless steel with a diameter of 200 mm, and the high voltage and ground electrodes were circular stainless-steel plates with a diameter of 50 mm and a thickness of 2 mm. The ground electrode was covered with a circular quartz glass dielectric sheet with a diameter of 100 mm and a thickness of 2 mm, and the treated material (the PP material selected in this study is a commercially available pure PP sheet characterized by high transparency and low density, which was processed into circular discs with a diameter of 70 mm and a thickness of 2 mm) was placed on the dielectric sheet with a fixed discharge gap of 1.5 mm and a treatment time of 1 min.

### 2.2. Diagnosis of DBD Plasma

In this experiment, Ar (purity 99.999%) and Ar/O_2_ mixture (99% Ar + 1% O_2_) were used as the working gases. The gas flow rate was controlled using a mass flow meter (Sevenstar D08-4F, Aurasky Electronics, Beijing, China) to adjust the proportion of O_2_ by controlling the ratio of pure Ar to O_2_-carrying Ar. The reactor chamber was pumped to a vacuum (5 Pa) using a pumping system, and the working gas was charged into the reactor to 1.01 × 10^5^ Pa at a rate of 1 L/min and held constant. The externally applied voltage and total current were measured using a high voltage probe (Northstar PVM-5, Northstar, WA, USA) and a current coil (Pearson Electronics Inc. 2877, Pearson Electronics, CA, USA). A 2.2 nF measuring capacitor (Cm) was connected in the series at the ground terminal, and the voltage signals (Um) on both sides of the Cm were measured using a differential probe (PinTech N1070Apro, Pintech, Guangzhou, China, 1000/100:1). Voltage–current waveforms were captured and stored using an oscilloscope (Tektronix 3054, Tektronix, OR, USA). Emission spectra were measured using an Ocean Optics HR4000CG-UV spectrometer (Ocean Optics, Shanghai, China, wavelength range 200 nm–1100 nm, resolution 0.75 nm). Discharge images were taken using a digital camera (Canon EOS 6D, Canon, Shanghai, China) with an exposure time of 50 ms.

### 2.3. Analysis Methods

Various analytical techniques were used to characterize the physical and chemical properties of the PP before and after plasma treatment. The surface morphology and roughness of the PP were examined using field emission scanning electron microscopy (SEM) (TESCAN MIRA4, TESCAN, Brno, Czech Republic) and atomic force microscopy (AFM) (Bruker Dimension^®^ Icon™, Billerica, MA, USA), operating in tapping mode. Before performing the SEM measurement, the samples were gold sputtered for 1 min with 15 mA sputtering current, and the metal layer thickness was about 10 nm. Additionally, the chemical composition of the PP before and after treatment was scrutinized using attenuated total reflectance Fourier-transform infrared spectroscopy (FTIR, Thermo NicoLet iS50, Thermo, Shanghai, China) and X-ray photoelectron spectroscopy (XPS, EscaLab 250Xi, Thermo, Shanghai, China). The spectral range for the FTIR-ATR analysis was set from 4000 to 400 cm^−1^, with a resolution of 4 cm^−1^ and 32 scans. For the XPS analysis, the power was set to 150 W, with a beam current of 10 mA and a beam spot size of 400 μm × 400 μm. The survey scan range was from 0 to 1200 eV for the surface element analysis, while high-resolution scans were performed for C1s, O1s, and other core-level spectra, with a resolution of approximately 0.5–1 eV. The modification effect and treatment uniformity were evaluated by the radial and fixed-point distribution of the water contact angle. The water contact angle was measured using a static water contact angle meter (ZJ-CAZ2, Z. Jia equipment, Shenzhen, China), with a 1.5 μL drop used for each measurement. To mitigate the influence of aging effects and to ensure accurate results, measurements were taken 5 h after treatment.

## 3. Results and Discussion

**Figure 2:**
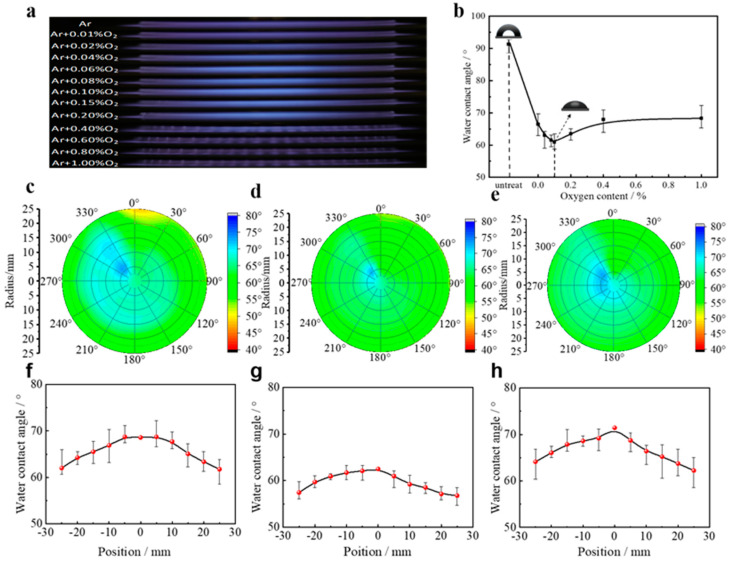
(**a**) Discharge images of nanosecond pulsed Ar DBDs under different O_2_ contents. (**b**) The water contact angle on the PP surface under various oxygen additions. Fixed-point distribution of water contact angle of (**c**) Ar, (**d**) Ar/O_2_ (0.1%), and (**e**) Ar/O_2_ (1%). Radial distribution of water contact angle of (**f**) Ar, (**g**) Ar/O_2_ (0.1%), and (**h**) Ar/O_2_ (1%).

In order to study the effect and homogeneity of Ar DBD on the surface treatment of PP materials under different O_2_ additions, the PP materials were modified under different O_2_ additions and the water contact angle on the surface of the materials was measured. The optimum content of O_2_ addition was determined by comparing the discharge characteristics and treatment effects. The discharge images of Ar DBD excited by nanosecond pulsed power supply under different O_2_ additions are shown in Figure 2a. It is evident that the overall discharge uniformity underwent a transition from uniform to inhomogeneous with increasing O_2_ additions. At lower O_2_ levels (0%~0.1%), the nanosecond pulse Ar DBD discharge maintained the stability and a higher degree of uniformity. This is attributed to the limited O_2_ content during this phase. The collision between high-energy electrons and oxygen molecules resulted in electron generation, which facilitated ionization through oxygen molecule collisions and promoted discharge development, thereby improving the overall uniformity. When the O_2_ addition was increased to 0.15%~0.2%, weak filaments began to appear at the edges of the discharge and the uniformity deteriorated. When the O_2_ addition was increased further (more than 0.2%), the discharge became completely filamentary, leading to further deteriorations in uniformity and the instability of the discharge. This is mainly because O_2_ is an electronegative gas. As the amount of O_2_ increased, the O_2_ molecules increasingly destabilized the Ar particles. A large number of electrons were absorbed by the oxygen molecules, reducing the number of electrons and stabilized particles available for ionization. This made the ionization of the working gas more difficult, leading to the transition to a filamentary discharge.

To assess the hydrophilicity of the modified PP materials, static water contact angle measurements were taken at ten random points on the PP samples, both before and after treatment. The average value derived from these measurements served as a representative indicator under the given conditions. Figure 2b shows the variation curves of the average water contact angle on the treated material surfaces for different O_2_ additions. The length of the error bars in the figure illustrates the scatter of the water contact angle values at the ten measurement points. Upon examination of the figure, it was observed that the water contact angle of the untreated PP material was 92.82°. As the O_2_ addition increased, the water contact angle initially decreased and then increased. Specifically, at 0.1% O_2_ addition, the water contact angle decreased from 66.47° to its minimum value of 60.94°, a reduction of 31.88° (52.31% decrease) compared to the untreated state. Further addition of O_2_ led to an increase in the water contact angle, with a slower overall upward trend. At 1% O_2_ addition, the average water contact angle was 68.31°. These results indicate that low O_2_ addition improves the surface hydrophilicity of the PP material, while excessive O_2_ compromises the modification effect. Notably, the difference between the maximum and minimum water contact angle values was minimal in the 0.08%–0.2% O_2_ addition range but increased significantly in the other O_2_ addition ranges. The correlation between the change in this difference and the discharge uniformity was pronounced, emphasizing that better discharge uniformity corresponded to a smaller disparity between the minimum water contact angle values.

To more intuitively characterize the hydrophilicity of the PP material’s surface after DBD plasma treatment, measurements were made using a polar coordinate system. The center of the sample was used as the pole, with rays drawn every 30°, and measuring points were taken every 5 mm along each ray. The water contact angle was measured at each point and different colors were used to represent different magnitudes, providing a clear visual representation of the water contact angle distribution across the material surface. The distribution of the water contact angle under different O_2_ additions is plotted in the polar coordinates shown in Figure 2c–e. At the same time, the water contact angles at different distances from the center of the material sheet after treatment were also measured, and their average values were calculated to obtain the variation curves of the average value of the water contact angle with the position of the radius under different O_2_ additions, as shown in Figure 2f–h.

**Figure 3:**
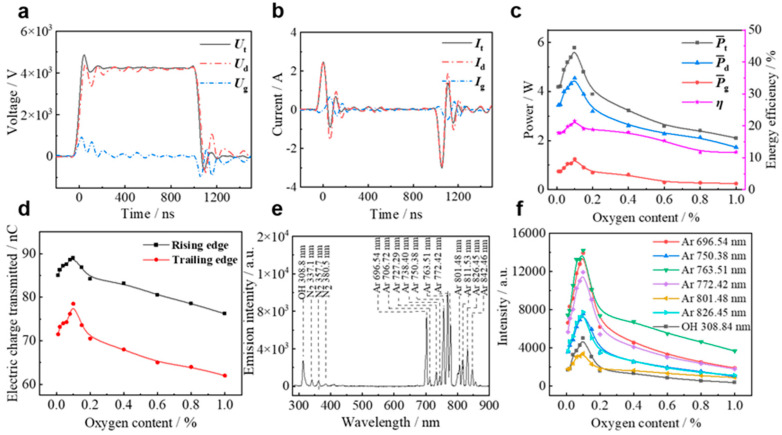
Results of the separation of (**a**) applied voltage *U*_t_ and (**b**) loop total current *I*_t_. (**c**) Trend of average power and energy efficiency at various oxygen additions. (**d**) Trend of transfer charge at various oxygen additions. (**e**) Typical emission spectra of the DBD under Ar conditions. (**f**) Trend of major particle intensities in Ar DBD emission spectra at various oxygen additions.

In addition to evaluating the modification effect at different O_2_ additions, it is important to understand the energy consumption during the modification process. In this paper, the voltage and current waveforms are measured and the relevant parameters are calculated [31]. By separating the waveforms of the externally applied voltage (*U*_t_) and the measured current (*I*_t_) during the discharge process, we derived crucial elements such as the voltage across the gap (*U*_g_), the voltage across the dielectric layer (*U*_d_), the displacement current (*I*_d_), and the conduction current (*I*_g_). From these electrical parameters, we calculated key metrics such as the power and energy efficiency. The computational and analytical procedures have been thoroughly investigated within our research group [32,33]. The separated voltage–current waveforms are shown in Figure 3a,b. In particular, during the rising phase of the voltage pulse from the nanosecond pulsed power supply, the increase in the *U*_t_ corresponds to an increase in the *U*_g_, thereby enhancing the spatial electric field. When the *U*_g_ was increased to the breakdown voltage, the gas gap broke down and *I_g_* appeared. The resulting plasma caused significant charge accumulation, reducing the impedance of the air gap and causing the *U*_g_ to rapidly decrease to 0. Due to the long lifetime of the space charge and surface charge generated by the discharge, a substantial charge accumulated on the dielectric plate during the falling phase of the voltage pulse. This resulted in a gradual decrease in the amplitude of the *U*_d_ and a reverse increase in the *U*_g_. When the *U*_g_ reached the breakdown voltage, a reverse discharge occurred, generating a counter-pulse current in the *I*_g_.

From the separate voltage and current measurements, the average power and energy efficiency of nanosecond pulsed Ar DBD under different O_2_ additions were calculated. This analysis provides the average power per discharge cycle, the average power in the gas gap, the average power in the dielectric layer, the energy efficiency (*η*), and the amount of charge transferred during the rising and falling edges of the pulse. Figure 3c shows the variation curves of the average power and energy efficiency with different O_2_ additions. The results revealed a distinct pattern: as O_2_ addition increased, both the average power and energy efficiency exhibited an initial increase followed by a subsequent decrease. Specifically, within the O_2_ addition range of 0% to 0.1%, the average power increased from 4.19 W to 5.79 W, and the *η* increased from 17.78% to 21.51%. However, when the O_2_ addition exceeded 0.1%, a general decrease in both the average power and energy efficiency was observed. The evaluation of the discharge strength and plasma chemical reaction rates, as measured by the transferred charge, is depicted in Figure 3d. Notably, the transfer charge at the rising and falling times showed an initial increase followed by a decrease with increasing oxygen addition. For example, in the O_2_ addition range 0% to 0.1%, the transfer charge at the rising and falling times increased from 85.04 nC and 71.52 nC at 0% to 89.10 nC and 78.55 nC at 0.1%, respectively. Conversely, when the O_2_ addition exceeded 0.1%, there was a decreasing trend in the transfer charge at the rising and falling times. At 1% oxygen addition, the transfer charge measured 76.21 nC and 62.05 nC. This suggests that optimal O_2_ addition enhanced the DBD discharge strength, injecting more energy into the discharge space and accelerating the chemical reaction rates. Further analysis indicates that within the range of 0% to 0.1% O_2_ addition, the limited O_2_ facilitates Penning ionization between the Ar metastable atoms and oxygen molecules, thereby promoting discharge. Conversely, above 0.1% O_2_ addition, an excess of O_2_ led to the adsorption of numerous electrons by the oxygen atoms, resulting in a reduction in the number of charged particles in the discharge space. This weakened the discharge intensity, which manifested itself in a significant decrease in the gas gap voltage, a reduction in the transmitted charge, a decrease in the average gas gap power, and a drop in the energy efficiency. These findings are consistent with the modification effects observed at different O_2_ additions.

The emission spectra serve as a valuable tool for revealing chemically active substances and characteristics within the plasma, offering crucial insights for analysis of the plasma reaction mechanism. Figure 3e displays the emission spectra of DBD under Ar conditions, with wavelengths mainly distributed in the range of 690~950 nm. Notably, nitrogen molecule spectral lines (337.1, 357.7, and 389.0 nm) and OH spectral lines (308.8 nm) also appeared due to trace impurity gases during discharges under Ar conditions. The variation of peak emission spectra of Ar DBD with an oxygen content ranging from 0% to 1% was analyzed, and the intensity trend of key particles in the emission spectra of Ar DBD under different O_2_ additions is depicted in Figure 3f. The plots revealed that the emission intensity of each spectral line followed an overall pattern of initial increase and subsequent decrease with rising O_2_ addition, reaching a peak at 0.1% O_2_ addition. The intensity of these spectral lines is proportional to the density of active particles in the discharge space. Consequently, Ar/O_2_ (0.1%) DBD generated a more reactive plasma with a higher concentration of active particles compared to Ar DBD. Further examination showed that when the O_2_ addition was less than 0.1%, oxygen molecules underwent excitation through collision ionization and Penning ionization between existing high-energy electrons and substable particles in the discharge space. This process promoted the generation of free electrons and resulted in an increased production of excited state Ar atoms in the discharge space. However, with an O_2_ addition above 0.1%, electrons were attached to electronegative oxygen molecules at a higher oxygen content. This limited the number of electrons involved in the collision and excitation of Ar and O_2_, increasing the difficulty of the air-gap breakdown, reducing the intensity of the DBD discharge, and reducing the overall spectral emission intensity.

SEM and AFM were used to analyze the surface morphology of untreated and treated PP surfaces. This analysis aimed to provide a deeper understanding of the modification mechanism under different conditions, including the numerical calculation of parameters such as surface roughness. *R*_q_ (root mean square roughness) is the standard deviation of the surface height, indicating the average degree of surface height fluctuation. *R*_a_ (arithmetic average roughness) is the arithmetic mean of the surface profile heights, representing the average deviation of the surface height from the mean plane. The calculation formulas are as shown in Equations (1) and (2):

(1) $$R_{a}=\frac{1}{n}\sum_{i=1}^{n}\left|z_{i}-\bar{z}\right|$$

(2) $$R_{q}=\sqrt{\frac{1}{n}\sum_{i=1}^{n}{\left(z_{i}-\bar{z}\right)}^{2}}$$

where *n* is the number of points within the measurement interval, and *z*_i_ is the distance between the height of the i-th point and the mean plane.

**Figure 4:**
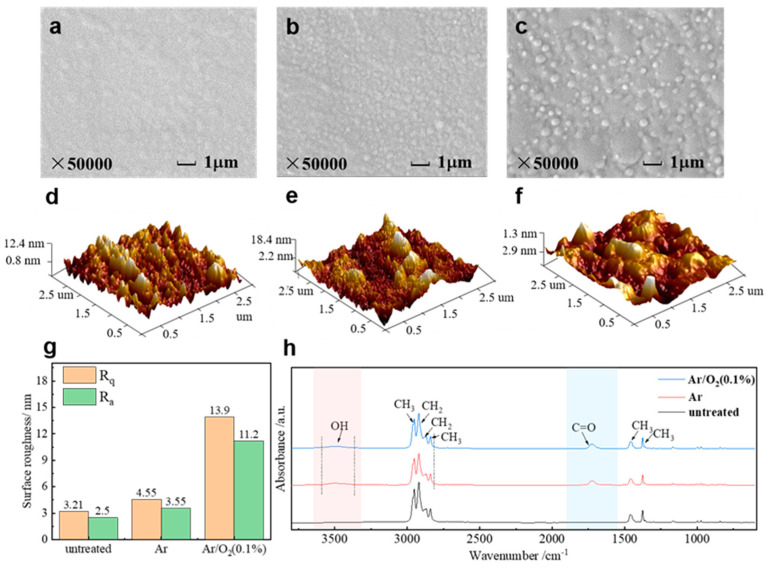
Surface morphology of PP surface at typical points by SEM of (**a**) untreated, (**b**) Ar, and (**c**) Ar/O_2_ (0.1%); 3D micromorphology of PP surface at typical points by AFM of (**d**) untreated, (**e**) Ar, and (**f**) Ar/O_2_ (0.1%). (**g**) The comparison of R_q_ and R_a_ of the material surface at various oxygen additions. (**h**) The ATR-FTIR spectra of polypropylene under different treatment conditions.

As can be seen from Figure 4a, the PP surface that did not undergo DBD modification treatment is relatively smooth, with only a few faint and insignificant scratches. Figure 4b displays the PP material surface after nanosecond pulsed Ar DBD treatment, illustrating the etching effect induced by the plasma treatment, which can alter the pore structure and surface roughness [34,35]. Figure 4c presents the microscopic image resulting from the addition of 0.1% O_2_ to Ar. Here, the material surface exhibits pronounced grooves created by the plasma etching effect, showcasing a superior degree and density compared to the previous image. The AFM images taken before and after the treatment are displayed in Figure 4d–f. The untreated PP surface exhibits a smoother profile with fewer grooves and variations in the three-dimensional images. In contrast, the post-nanosecond pulse Ar DBD treatment revealed deep grooves on the PP surface. When comparing the Ar DBD with the Ar/O_2_ DBD, the latter showed a higher density and average power of the active particles in the discharge space, resulting in more significant bombardment and etching on the PP material surface. As a result, the grooves on the Ar/O_2_ DBD treated surface are deeper and denser, explaining the greater reduction in the water contact angle and the more uniform hydrophilicity observed after treatment.

**Figure 5:**
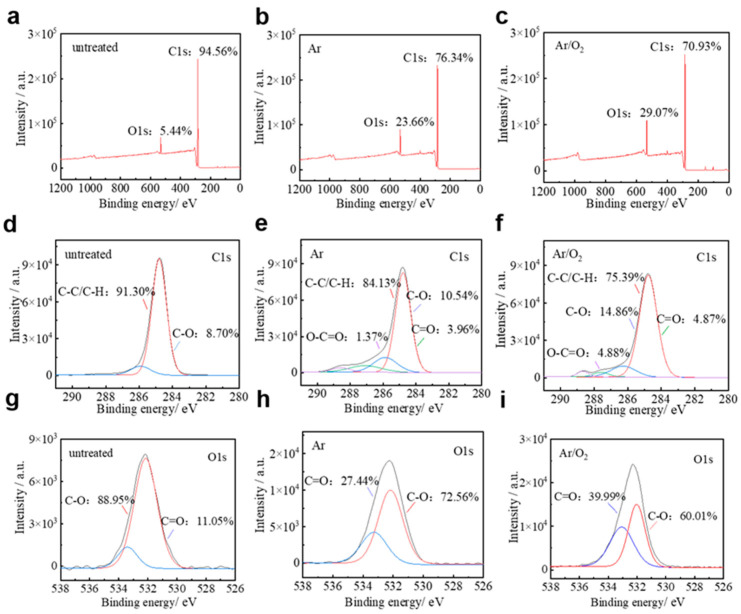
XPS spectrum of (**a**) untreated, (**b**) Ar DBD-treated, and (**c**) Ar/O_2_ (0.1%) DBD-treated, C1s spectra of (**d**) untreated, (**e**) Ar DBD-treated, and (**f**) Ar/O_2_ (0.1%) DBD-treated. O1s spectra of (**g**) untreated, (**h**) Ar DBD-treated, and (**i**) Ar/O_2_ (0.1%) DBD-treated.

To analyze the effect of the DBD modification treatment on the chemical composition of the PP materials, FTIR and XPS chemical composition tests were conducted on both the untreated and treated PP surfaces. Figure 4h shows a comparison of the ATR-FTIR spectra after treatment under different conditions. The untreated PP spectrum exhibited four peaks in the wave number range of 3000 cm^−1^ to 2800 cm^−1^, corresponding to various CH_3_ and CH_2_ vibrational modes. The DBD treatments introduced new absorption peaks, particularly between 3620 cm^−1^ and 3300 cm^−1^ (attributed to OH vibration) and at 1738 cm^−1^ (attributed to C=O vibration). These peaks indicate the incorporation of oxygenated hydrophilic groups, including -OH and C=O, on the PP material surface due to the DBD modifications. The appearance of C=O and OH-related peaks in the FTIR spectra of the samples treated with only Ar is primarily due to the evacuation of the reactor to a pressure of 5 Pa using a vacuum system. Due to technical limitations, it was not possible to achieve an absolute vacuum (0 Pa). Therefore, during the plasma treatment of polypropylene (PP), a small amount of air, including oxygen and water molecules, remained in the discharge chamber. The plasma treatment excited the oxygen molecules in the air, generating highly reactive oxygen species (such as atomic oxygen and hydroxyl radicals) that react with the PP surface, leading to the formation of C=O and OH functional groups. Specifically, the oxidation reaction leads to the formation of carbonyl (C=O) and hydroxyl (OH) groups. The carbonyl groups are formed when the carbon atoms on the PP surface react with reactive oxygen species to generate oxygenated compounds such as aldehydes and ketones, while the hydroxyl groups are formed through reactions with hydroxyl radicals. The above reactions are reflected in the FTIR spectrum by a C=O absorption peak around 1738 cm^−1^ and an OH absorption peak in the range of 3300–3620 cm^−1^, while in the XPS spectrum (Figure 5b) they are indicated by an increase in the oxygen content. Notably, despite these changes, the main peak shapes and positions in the ATR-FTIR spectra remain relatively consistent under different treatment conditions. This suggests that the plasma modification treatment does not compromise the chemical structure of the PP material, but selectively alters the chemical composition of the material surface.

XPS was used to quantitatively analyze the chemical elements present on the PP surface before and after treatment. The chemical element content is shown in Table 1. The O1s and C1s peaks in Figure 5a were found to have C and O elemental proportions of 94.56% and 5.44%, respectively, on the unmodified treated PP surface, with binding energies of 284.80 eV and 532.10 eV, respectively. Elemental O increased significantly to 23.66% after nanosecond pulsed Ar DBD treatment, and to 29.07% after nanosecond pulsed Ar/O_2_ DBD treatment. These increases indicate that many oxygen-containing groups have been introduced to the surface of the treated material. To determine the group composition and relative content of the corresponding elements, narrow-spectrum scans were conducted on the C1s and O1s peaks. The detection data are then split-peak fitted. The C1s peak of the treated PP material can be split into four different peaks corresponding to the C-C and C-H bonds (~285.00 eV), C-O bonds (~286.50 eV), C=O bonds (~287.70 eV), and O-C=O bonds (~289.10 eV) [36]. The bond energies of the C-C and C-H bonds in PP materials are 3.6 eV and 4.3 eV, respectively [37]. The constant collisional ionization of the high-energy electrons (1–10 eV) can effectively break the C-C and C-H bonds, leading to bond breakage. It is evident that after treatment, the content of the C-H groups on the PP material decreases, while the content of the C-O, C=O, and O-C=O groups increases. This suggests the opening of C-C/C-H bonds, with the continuous implantation of oxygen-containing groups such as C-O, C=O, and O-C=O onto the surface of the PP material through reactions. The introduction of these oxygen-containing groups increases the polarity of the PP materials and the polar molecules have a high affinity for water, which can attract the water molecules, thus improving the hydrophilicity. As can be seen in Figure 5g, the O1s peak of unmodified treated PP also consists of two peaks, C-O and C=O, superimposed on each other, and the relative content of hydrophilic groups of C=O is increased after nanosecond pulsed Ar DBD and Ar/O_2_ DBD treatments, respectively, which indicates that the modification treatments increase the proportion of hydrophilic groups on the surface of the PP materials.

The relative content of each constituent group was obtained from the results of the split-peak fitting of the C1s and O1s peaks, and the C1s peak of the untreated PP in Figure 5d consists of two peaks of C-C/C-H and C-O superimposed on each other, and the relative contents of the two are 91.30% and 8.70%. This is shown in Table 2. In Figure 5e, two new peaks corresponding to the C=O and O-C=O appear on the surface of the treated PP material. Additionally, the C-C/C-H ratio decreases from 91.30% in the untreated sample to 84.13%, while the C-O content increases to 10.54%. The newly formed oxygen-containing groups, C=O and O-C=O, are 3.96% and 1.37%, respectively. The proportions of oxygen-containing groups C=O and O-C=O further increase, as shown in Figure 5f, while the C-C/C-H ratio decreases to 75.39%. Specifically, the peaks associated with C-O and O-C=O may overlap between the C1s and O1s regions. This means that the relative intensities of these components can differ when fitted in the C1s region versus the O1s region, due to differences in how the overlapping signals are decomposed and assigned to each element. As a result, the relative amounts of C=O, C-O, and O-C=O in the C1s spectrum might not be directly consistent with those in the O1s spectrum. The results show that the pure Ar modification treatment increases the proportion of oxygen-containing groups on the surface of the PP materials, and the addition of an appropriate amount of O_2_ can further increase the proportion of oxygen-containing groups. The introduction of oxygen-containing groups increases the molecular polarity, which improves the surface hydrophilicity of the PP materials.

**Figure 6:**
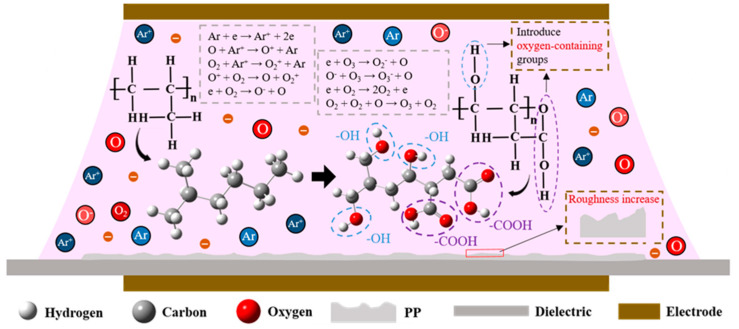
Illustration of the PP surface modification process using Ar/O_2_ DBD.

Figure 6 shows the molecular structure of PP and the process by which C-H bonds are opened and oxygen-containing groups are introduced during DBD processing. Under the action of a high-voltage electric field, the Ar in the DBD discharge space is ionized, generating a large number of high-energy electrons and abundant excited-state particles. These reactive particles bombard the PP surface, increasing the roughness of the material. At the same time, these particles break the molecular chemical bonds (C-C and C-H bonds) and introduce oxygen-containing groups (-OH, C=O, O-C=O) to the surface of the material, thus increasing the surface hydrophilicity. In addition, the addition of an appropriate amount of O_2_ enhances collisional ionization and increases the number of active particles. Compared to the pure Ar conditions, when moderate amounts of O_2_ are added the degree of surface etching is relatively consistent, the overall chemical bonds on the surface are opened uniformly, and hydrophilic groups are introduced at various locations. As a result, the material achieves greater surface roughness and a more evenly distributed arrangement of hydrophilic groups, facilitating uniform surface modification.

## 4. Conclusions

This study investigated the effect of varying O_2_ additions on PP surfaces subjected to nanosecond Ar DBD pulses. Using the DBD equivalent model, electrical parameters were calculated, discharge uniformity was assessed by images, and chemically active plasma features were unveiled by emission spectra. The interplay between discharge uniformity and material modification uniformity was explored using water contact angle measurements. SEM, AFM, FTIR, and XPS were employed for comprehensive characterization of the physical morphology and chemical composition. The results showed that a small addition of O_2_ to Ar not only increases the discharge strength, but also increases the number of oxygen-containing groups, which enhances the modification effect. Compared to the untreated material, a 0.1% O_2_ addition reduces the average water contact angle on the PP surface by 52.31%, reaching a minimum value of 60.94°. The distribution of water contact angles becomes more uniform within the plane, but this hydrophilic modification effect diminishes beyond a 0.1% addition. The SEM and AFM physical morphology analyses revealed that the addition of O_2_ caused the grooves produced by plasma etching to cover the entire material surface more extensively than when no O_2_ was added. The FTIR and XPS tests showed that the addition of O_2_ contributes to a more uniform oxygenated polarization, introducing oxygen-containing polar groups (-OH, C=O, and O-C=O). While this enhances the surface hydrophilicity, it is insufficient to achieve complete hydrophilic modification. This study shows that the increase in surface roughness and the introduction of oxygen-containing polar groups together increase the hydrophilicity of the material’s surface. Therefore, the addition of an appropriate amount of O_2_ to Ar proves to be an effective approach for the modification effects of DBD treatment on materials.

## Figures and Tables

**Table 1:** Surface chemical element composition of PP materials after treatment under different conditions.

Conditions	C	O	O/C
Untreated	94.56%	5.44%	5.75%
Ar DBD-treated	76.34%	23.66%	31.00%
Ar/O_2_ (0.1%) DBD-treated	70.93%	29.07%	40.98%

**Table 2:** Surface chemical composition of PP materials after treatment under different conditions.

Conditions	C1s				O1s	
	C-C/C-H	C-O	C=O	O-C=O	C-O	C=O
Untreated	91.30%	8.70%	-	-	88.95%	11.05%
Ar DBD-treated	84.13%	10.54%	3.96%	1.37%	72.56%	27.44%
Ar/O_2_ (0.1%) DBD-treated	75.39%	14.86%	4.87%	4.88%	60.01%	39.99%

## Data Availability

The original contributions presented in this study are included in the article. Further inquiries can be directed to the corresponding authors.
